# Engineering Lateral Heterojunction of Selenium‐Coated Tellurium Nanomaterials toward Highly Efficient Solar Desalination

**DOI:** 10.1002/advs.201900531

**Published:** 2019-08-17

**Authors:** Chenyang Xing, Dazhou Huang, Shiyou Chen, Qichen Huang, Chuanhong Zhou, Zhengchun Peng, Jiagen Li, Xi Zhu, Yizhen Liu, Zhipeng Liu, Houkai Chen, Jinlai Zhao, Jiangqing Li, Liping Liu, Faliang Cheng, Dianyuan Fan, Han Zhang

**Affiliations:** ^1^ Shenzhen Engineering Laboratory of Phosphorene and Optoelectronics International Collaborative Laboratory of 2D Materials for Optoelectronics Science and Technology of Ministry of Education College of Physics and Optoelectronic Engineering Shenzhen University Shenzhen 518060 China; ^2^ Center for Stretchable Electronics and Nanoscale Systems Key Laboratory of Optoelectronic Devices and Systems of Ministry of Education College of Physics and Optoelectronic Engineering Shenzhen University Shenzhen 518060 China; ^3^ College of Chemistry and Environmental Engineering Shenzhen University Shenzhen 518060 China; ^4^ School of Science and Engineering The Chinese University of Hong Kong Shenzhen 518172 China; ^5^ Shenzhen Institute of Artificial Intelligence and Robotics for Society Shenzhen Guangdong 518172 China; ^6^ Nanophotonics Research Center Shenzhen Key Laboratory of Micro‐Scale Optical Information Technology Shenzhen University Shenzhen 518060 China; ^7^ Faculty of Information Technology Macau University of Science and Technology Avenida Wai Long Taipa Macau 999078 China; ^8^ Department of Hepatobiliary and Pancreatic Surgery Shenzhen People's Hospital Second Clinical Medical College of Jinan University Shenzhen 518060 China; ^9^ Dongguan University of Technology Dongguan 523808 China

**Keywords:** heterojunctions, selenium, solar desalination, tellurium

## Abstract

Herein, a core–shell tellurium–selenium (Te–Se) nanomaterial with polymer‐tailed and lateral heterojunction structures is developed as a photothermal absorber in a bionic solar‐evaporation system. It is further revealed that the amorphous Se shell surrounds the crystalline Te core, which not only protects the Te phase from oxidation but also serves as a natural barrier to life entities. The core (Te)–shell (Se) configuration thus exhibits robust stability enhanced by 0.05 eV per Se atom and excellent biocompatibility. Furthermore, high energy efficiencies of 90.71 ± 0.37% and 86.14 ± 1.02% and evaporation rates of 12.88 ± 0.052 and 1.323 ± 0.015 kg m^−2^ h^−1^ are obtained under 10 and 1 sun for simulated seawater, respectively. Importantly, no salting out is observed in salt solutions, and the collected water under natural light irradiation possesses extremely low ion concentrations of Na^+^, K^+^, Ca^2+^, and Mg^2+^ relative to real seawater. Considering the tunable electronic structures, biocompatibilities, and modifiable broadband absorption of the solar spectrum of lateral heterojunction nanomaterials of Te–Se, the way is paved to engineering 2D semiconductor materials with supporting 3D porous hydrophilic materials for application in solar desalination, wastewater treatment, and biomedical ventures.

Semiconductor nanomaterials that are based on photothermal agents have drawn considerable attention owing to their ability to convert light into heat energy, for practical applications, such as in the photothermal therapy of cancer^1^, ^2^, ^3^, ^4^, ^5^ and solar evaporation for water purification.^6^, ^7^, ^8^, ^9^ In solar evaporation, photothermal agents must satisfy several requirements. First, they should absorb sunlight and convert it to heat to evaporate water. Hence, sufficient light absorption over the entire solar spectrum (280–2500 nm) is desirable.^10^ Semiconductors with indirect/direct and tunable energy bandgaps can absorb light over a broad range of wavelengths, which may contribute to good photothermal conversion efficiencies.^11^ Second, photothermal materials should be biocompatible and nontoxic because of their potential large‐scale use close to drinking water.^12^ Inorganic photothermal agents are typically used in composites for solar‐evaporation applications. These structures are typically 3D porous skeletons, such as hydrogels,^13^ sponges,^14^ tree stumps,^15^ and flat filter papers^16^, ^17^ or membranes.^7^ There are two main advantages of such composite materials. First, well‐dispersed photothermal agents can transform light to heat more effectively than compacted agents; thus, a better dispersal of the agents can reduce material use and costs. Second, the composite can enable a continuous sustained water supply for evaporation. In water‐evaporation applications, an important issue to be overcome is salt crystallization onto the 3D mesh of the composite materials.^14^, ^16^ These crystals can reflect sunlight, reduce light absorption, and decrease the photothermal conversion efficiency. Salt crystallization–induced inefficiencies are determined by the 3D structure of the mesh. Other desirable properties for practical applications of photothermal materials include hydrophilic surfaces, a good chemical stability, the ability to adhere to 3D skeletons in the presence of water, and the potential for large‐scale application. Besides the photothermal agents, reasonable designs of evaporation systems are important to meet highly efficient solar‐desalination performance, such as the use of thermal concentration,^18^ interfacial solar steam generation,^19^ and enhancing solar absorption and thermal conversion.^20^

Recently, tellurium (Te) has emerged as a new 2D material with a broad *E*_g_ and excellent optoelectronic properties.^21^, ^22^, ^23^, ^24^, ^25^ Ye's group reported a large‐scale application of 2D Te nanosheets that were generated via a hydrothermal route from Te source.^21^ He's group fabricated 2D Te by a van der Waals epitaxy approach.^23^ In our previous study, we fabricated small 2D Te nanosheets by liquid‐phase exfoliation.^22^ Although Te has a unique electronic structure, which enables broadband absorption of sunlight, there have been few investigations of Te‐based photothermal evaporation systems. Recently, Yang's group reported on Te‐based nanoparticles for solar evaporation.^26^ The Te in their study was plasmonic‐like and had all dielectric properties in the solar‐radiation region, which suggests potential for Te‐based photothermal conversion materials to be used in water evaporation. Unfortunately, the long‐term biocompatibility and biosafety of Te nanoparticles were not studied.^27^ Furthermore, the photothermal performance of the Te nanoparticles has only been reported with pure water and not seawater, which is a more practical target. The effects of salt crystallization have yet to be investigated in this material system. These issues are of great importance for the use of Te‐based photothermal agents in solar‐evaporation applications. Te is a toxic element and harmful waterborne pollutant, which may threaten human and aquatic life. To overcome this drawback, additional chemical treatments should be conducted to minimize the Te toxicity and enhance its long‐term stability against oxidation or degradation while maintaining its advantages, such as a broadband light absorption, a high photothermal efficiency, and a high electron mobility.

We report 3D composite sponges, based on selenium (Se)‐coated Te (Te–Se) nanomaterials and commercial melamine sponge (MS), for highly effective solar desalination. The Te–Se nanomaterials were synthesized by a hydrothermal route and coated onto the MS 3D skeletons. Unlike Te, which has toxic degradation products, the Te–Se nanomaterials with core (Te)–shell (Se) nanostructures (actually lateral heterojunction structures) showed an excellent stability and photothermal properties. Furthermore, their biocompatibility and biosafety were demonstrated through in vivo and in vitro testing. The 3D melamine support was pretreated physically with poly(diallyldimethylammonium chloride) (PDDA) in water to form PDDA@MS. The as‐prepared Te–Se nanomaterials adhered robustly to the 3D skeletons for long‐term applications. The as‐prepared Te–Se@PDDA@MS exhibited highly efficient solar desalination with a stable energy efficiency greater than 90% in water and NaCl and MgCl_2_ solutions without any salt crystallization. Our approach offers a route to developing biocompatible Te–Se‐based materials and provides a versatile strategy to develop inorganic/sponge‐based photothermal solar‐evaporation materials for potential industrial applications.

Because they are located in the same main group (chalcogens) in the periodic table, Te‐ and Se‐based crystals have similar features, such as their ability to form chain‐like structures, their indistinguishable crystal‐cell parameters, and their wide‐spectrum absorption. It is reasonable to assume that Te‐based nanostructures could be modified chemically by Se atoms or a Se phase, and vice versa, to form new Te–Se‐based nano‐heterojunctions with intriguing morphologies and physical properties that differ from those of the Te‐ and Se‐only‐based nanomaterials. In this study, a common hydrothermal reaction strategy was used to produce Te–Se‐based heterojunction nanostructures. In particular, the reduction of Te from its precursor Na_2_TeO_3_ was conducted in the presence of polyvinylpyrrolidone (PVP) ligand and Na_2_SeO_3_. Therefore, our Te–Se nanomaterials are composed of Te phase, Se phase, and PVP component, and their weight percent values can be obtained by using inductively coupled plasma atomic emission spectroscopy (ICP‐AES), as shown in Table S1 (Supporting Information). **Figure** **1** shows typical morphologies of Te‐ and Te–Se‐based nanomaterials. Neat Te nanowires of several micrometers in length and a few nanometers in diameter, without Se addition, are shown in Figure 1a. Such a 1D nanowire‐shaped Te morphology is commonplace in the literature, and is largely originated from its chain‐like structure of Te crystals.^28^ Interestingly, much shorter and wider oval‐like morphologies exist in the case of Te–Se samples, as shown in Figure 1b–d. For instance, with a molar ratio of Te to Se of 1/0.25 (namely, an initial precursor ratio) in Figure 1b, compared with Te nanowires, the Te–Se (1/0.25) sample shows a major axis (also termed a lateral dimension) of ≈40–110 nm and a minor axis of ≈35–50 nm, respectively. In addition, with an increase in Se content, resultant Te–Se nanomaterials with ratios of 1/0.5 (Figure 1c) and 1/1 (Figure 1d) are observed with gradually decreased dimensions in the major and minor axes. The clear changes in Te morphology suggest that Se addition helps to alter the Te growth dynamics. The Te–Se nanomaterial components were studied by using elemental mapping analysis, as shown in Figure 1e–l. Te–Se (1/0.25) can be detected as Te (Figure 1e) and Se (Figure 1f) signals, respectively. The Te‐rich accumulated image of a single particle is compact and distinguishable (Figure 1e), whereas the counterpart of Se (Figure 1f) is relatively scattered, loose, and larger in volume. This phenomenon may imply that the Te phase has a good crystallinity and that the Se phase is less crystalline in the Te–Se nanocomposites. The integration of these two elements in Figure 1g shows the coverage of the Te phase with an outer larger Se phase, which suggests a typical core (Te)–shell (Se) nanostructure. The O elemental signal is also detected, shown in Figure 1h, which is attributed to the PVP chains. A similar observation is found for Te–Se (1/1), shown in Figure 1i–l. A high‐resolution transmission electron microscopy (TEM) image (Figure 1m) of the Te–Se (1/1) sample shows a clear crystal lattice of ≈0.276 nm on the surface and also shows an amorphous or less‐crystalline part at the edge (shown by the arrows). The crystalline part results from the Te phase and the less‐crystalline part is from the Se phase. Therefore, the Te core is crystallized but its Se shell is less crystalline. The thickness of the Te–Se samples was evaluated by using atomic force microscopy (AFM), as shown in Figure 1n,o. For instance, as for the case of Te–Se (1/1), its measured thickness is ≈20–30 nm, as shown in Figure 1p. According to the above analysis, the Te phase, Se phase, and PVP chains contribute to the thickness.

**Figure 1:**
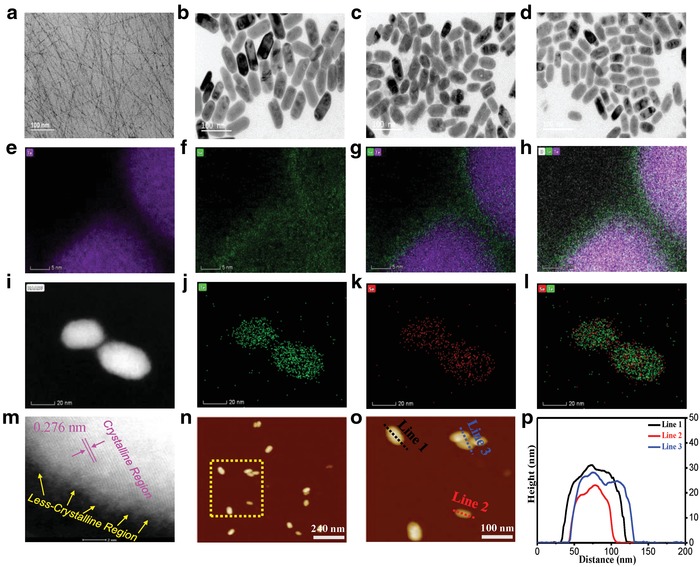
TEM images (including elemental distribution images), AFM images, and the corresponding height line profiles for Te‐ and Te–Se‐based nanostructures. TEM images: a) neat Te [Te–Se (1/0)], b) Te–Se (1/0.25), c) Te–Se (1/0.5), and d) Te–Se (1/1); elemental distribution images of Te–Se (1/0.25): e) Te‐rich element image, f) Se‐rich element image, g) integrated Te–Se element image, and h) integrated Te–Se–O element image; elemental distribution images of Te–Se (1/1): i) two individual particles, j) Te‐rich element image, k) Se‐rich element image, and l) integrated Te–Se element image; HRTEM image: m) Te–Se (1/1); AFM images: n,o): Te–Se (1/1); p) height profiles obtained from (o).

To understand the physical structure of the Te–Se nanomaterials, X‐ray diffraction (XRD) patterns of commercial bulk Te crystal powder, bulk Se crystal powder, and the Te–Se with various ratios of Te to Se were recorded, as shown in **Figure** **2**a. Similar crystal diffraction peaks of bulk Te and Se were observed (Figure 2a), such as peaks at 23.0°, 29.9°, 40.4°, 43.3°, 45.9°, 51.9°, and 65.9° for bulk Te (as shown by the red frame) and the corresponding peaks at 23.6°, 29.8°, 41.4°, 43.8°, 45.5°, 51.8°, and 65.3° for bulk Se (as shown by the black frame), which demonstrates their similar crystal parameters. Such a similarity may be a prerequisite for modifying Te crystals with a Se phase. The crystal diffraction patterns of Te–Se samples with redshift peaks are more similar to that of bulk Te crystals and they are indistinguishable from those of bulk Se crystals. Such a phenomenon supports that the Te phase is crystalline and that the Se phase is less crystalline in the Te–Se samples, which agrees well with our above TEM results. The shifted peaks of the Te–Se samples, relative to bulk Te, may be attributed to lattice strain that originated from the induction of amorphous‐like Se‐atom dopants.

**Figure 2:**
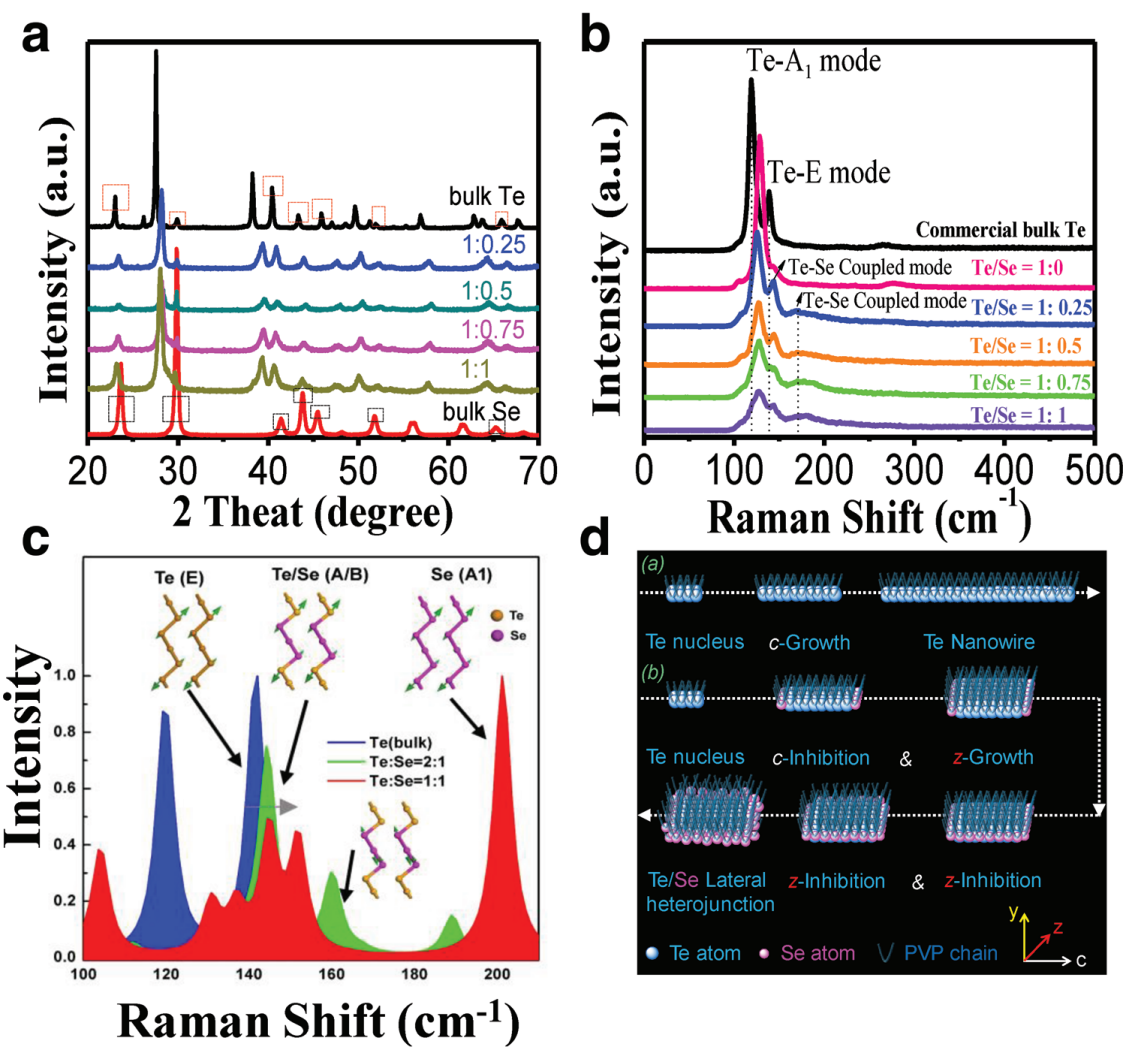
Characterization and simulation of Te–Se nanomaterials and their counterparts, such as bulk Te crystal, bulk Se crystal, and neat Te nanowires. a) XRD pattern from 20° to 70°. b) Raman spectra at an excitation wavelength of 633 nm. c) Simulated Raman spectra. d) Proposed synthesis mechanism of 1D Te nanowire and 2D Te–Se nanomaterials in the presence of PVP ligand.

Two typical peaks at 119.0 and 138.8 cm^−1^ in the commercial bulk Te (Figure 2b) are assigned to the A_1_ and E modes of Te, respectively.^22^, ^29^ For the neat Te nanowires [i.e., Te–Se (1:0)], the A_1_ mode of Te was clearly shifted to a higher energy, which we attribute to confinement effects in the nanosized Te nanowires in diameter. For the Te–Se nanomaterials, we found that as the Se content increased, the peak intensity of the A_1_ mode of Te and the coupled modes from the E mode of Te and the A_1_ mode of Se (as arrows in Figure 2b) decreased and the half peak width broadened. The absence of a main vibration peak of the bulk Se crystal, reported at ≈236 cm^−1^ in our previous study,^30^ indicates that the Se phase in the Te–Se nanomaterials is much more amorphous. The above Raman results support the above TEM and XRD results indirectly. Theoretical calculations of the Raman spectrum for bulk Te and Te–Se showed that higher‐energy Se–Te chemical bonds that formed at the interface enhanced the vibrational modes (Figure 2c), which may explain the changes in intensity and half peak width of the A_1_ and E modes of Te. The broadening phenomenon of these modes suggests that more discrete vibrational modes exist in the samples and the Se elements are not distributed homogeneously around the Te cores.

Te–Se nanomaterials are composed of a crystalline Te core, an amorphous Se shell, and tailed polymer PVP chain segments. Such an intriguing nanostructure may be understood from the crystal nucleation and growth perspective. Figure 2d presents possible mechanisms of the formation of 1D Te nanowires and Te–Se nano‐heterojunction structures. PVP can act as a crystal‐face‐blocking ligand to absorb Te atoms to form Te–PVP‐based crystal cells.^21^ Owing to the chain‐like structure of the Te, preferential growth occurs along the (001) plane, namely, the *c*‐axis of these crystal cells with an increase in reaction time, which results in a typical 1D Te morphology (several micrometers in length and few nanometers in diameter). However, when the Se precursors (Na_2_SeO_3_) are present in the reaction system, the Te crystal growth process changes. Te has a more rapid reduction speed from TeO_3_^2−^ anions to Te atoms than that of Se. Thus, it is assumed that in the redox reaction system, Te atoms emerged first and Se atoms second. Because of their similar crystal lattice, the Se atoms arranged first into Te crystals along the end of the (001) plane, which may hinder the length direction growth (*c*‐inhibition). Subsequently, when the Te crystals grew in the short‐axis direction to increase the short‐axis dimension, Se atoms around them began their second hindering process (*z*‐inhibition). An increase in reaction time can increase the outer Se shell thickness. Such a growth procedure gives rise to a Te (core)–Se (shell) lateral heterojunction. The PVP ligand chains can cause a second coating layer on the Te–Se surfaces, which helps the strong adherence onto a 3D skeleton (shown below).

Effective solar absorption and robust chemical stability of the photothermal materials are of great significance in sea solar desalination for long‐term usage. **Figure** **3** presents relevant characterizations of neat Te nanowires [Te–Se (1/0)] and Te–Se nanomaterials. The neat Te‐nanowire solution, which was prepared by a hydrothermal reaction method, is dark blue (Figure 3a), and its corresponding typical optical absorption is observed in a broad‐spectrum manner, as shown in Figure 3b (day 1). The maximum absorption is located in the visible‐light region, and is accompanied with good short‐wavelength near‐infrared absorption. Such a favorable optical absorption implies that neat Te may be an ideal candidate for solar photothermal materials. Unfortunately, neat Te nanowires degraded after a few days (Figure 3a), and the resultant solution became colorless. The gradual fading of the color of the water for the Te nanowire can be reflected by the gradual decrease in absorbance (Figure 3b). Additionally, such a degradation trend cannot be avoided in 3.5 wt% NaCl solution, as shown in Figure 3c. In sharp contrast, Te–Se nanomaterials, with the introduction of a Se phase, showed a considerable improvement in chemical stability. First, a broad absorption range was observed for all Te–Se samples with various Te to Se ratios (Figure 3d). The maximum absorption was found in the ultraviolet (UV) region. Obvious changes in optical absorption between Te‐only and Te–Se samples were ascribed to the variety of energy bandgaps (*E*_g_) from the introduction of a Se phase within the Te matrix. The Te–Se samples showed a robust chemical stability. For example, for the Te–Se (1/1) case, its light‐yellow color remained unchanged after days (Figure 3a). The corresponding absorption intensity in water was also almost unchanged (Figure 3e). Such a good stability can be retained in 3.5 wt% NaCl solution, with a slight decrease in peak value (Figure 3f). Te nanowire can degrade to high‐valency cations, probably TeO_3_^2−^, by detecting its 30‐day degradation production (Figure 3g).

**Figure 3:**
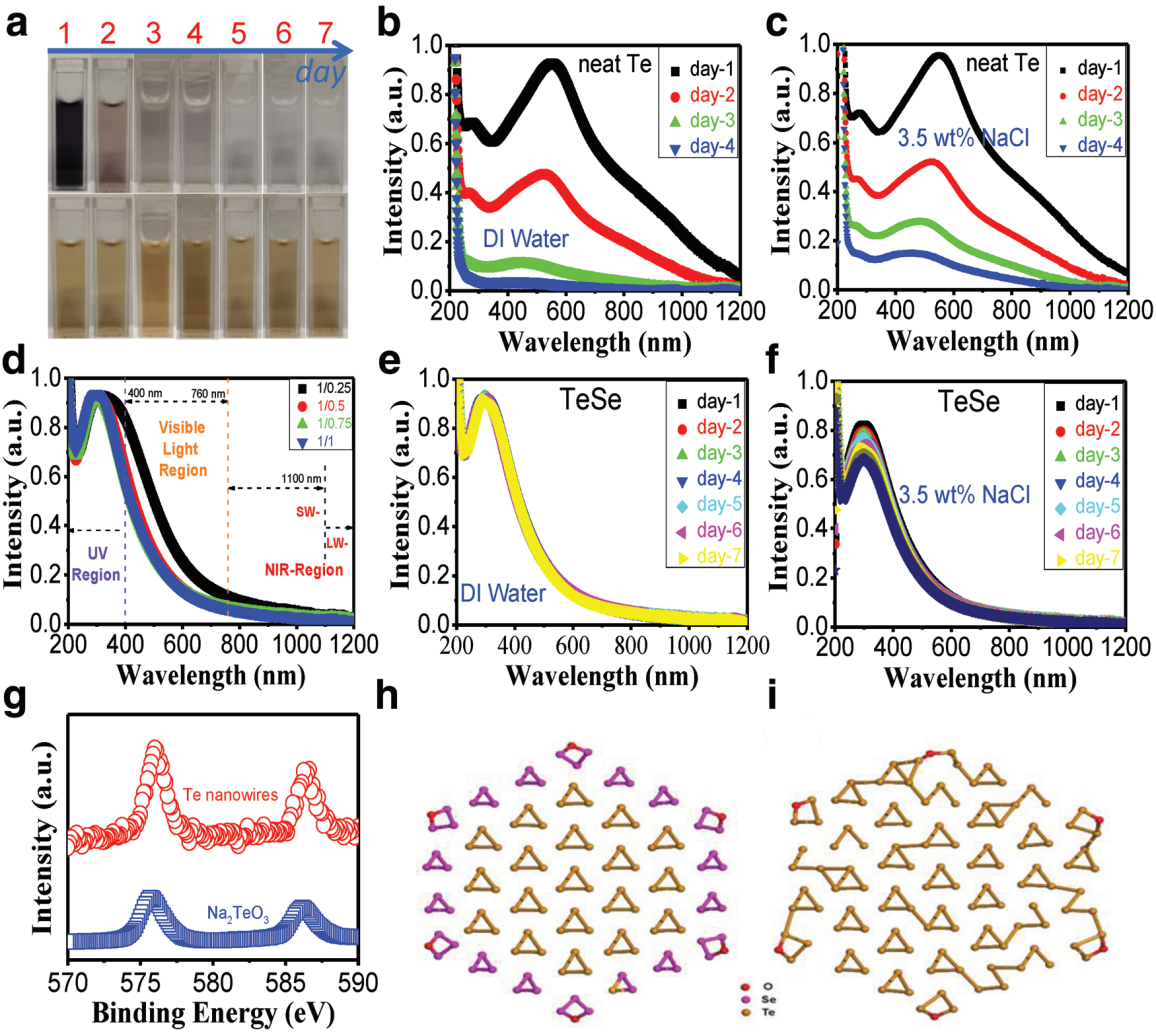
Optical and stability characterization of Te–Se nanomaterials and neat Te nanowires. a) photographs of Te‐only nanowires (up) and Te–Se (1/1) sample (down) with a 7‐day time observation. The first day is the day that the samples have been dialyzed. UV–vis absorption spectra for 200–1200 nm; solvent: DI water or 3.5 wt% NaCl solution: b) neat Te nanowire, for 4 days in DI water; c) neat Te nanowire, for 4 days in 3.5 wt% NaCl solution; d) Te–Se (1/0.25, 1/0.5, 1/0.75, and 1/1) nanomaterials in DI water; e) Te–Se (1/1) sample, lasting for 7 days in DI water; f) Te–Se (1/1) sample, lasting for 7 days in 3.5 wt% NaCl solution; g) XPS spectra: Te 3d; DFT optimized atomic structures for the oxides of h) Te–Se hybrids (Te_57_Se_54_O_6_) and i) Te cluster (Te_111_O_6_) (red, pink, and orange represent oxygen (O), selenium (Se), and tellurium (Te), respectively).

To investigate the shell‐Se‐induced stability in the Te–Se hybrid compounds and the pure Te cluster, we calculated the ground state of the two atomic structures, as shown in Figure 3h,i. Their chemical formulas are Te_57_Se_54_O_6_ (Figure 3h) and Te_111_O_6_ (Figure 3i). The optimized structures show that the shell Se layer can preserve the core structures from oxidation compared with pure Te layers, because of the relative stronger Se—O bonding energy compared with the Te—O bonding energy. The binding energy difference between Te_57_Se_54_O_6_ and Te_111_O_6_ $\left[i.e.,\;\;\Delta E=E^{{\mathrm{Te}}_{111}O_{6}}\;-E^{{\mathrm{Te}}_{111}}-6E^{O}-\left(E^{{\mathrm{Te}}_{57}{\mathrm{Se}}_{54}O_{6}}-E^{{\mathrm{Te}}_{57}{\mathrm{Se}}_{54}}-6E^{O}\right)\right]$ is calculated to be ≈0.05 eV per Se atoms, which indicates an enhancement of the stability, thanks to the increase in Se/Te ratio from 0:1 (Figure 3i) to ≈1:1 (Figure 3h).

Ideal photothermal conversion materials should be eco‐friendly, especially in the water‐treatment field. We thus examined the toxicity of neat Te and Te–Se‐based nanomaterials in vitro and in vivo. The neat Te nanowires [i.e., Te–Se (1/0) sample] were toxic toward three cell lines, including 3T3 (**Figure** **4**a), A549 (Figure 4b), and HCT116 (Figure 4c). The A549 cell line is more susceptible to Te‐only nanowires than the other two detected lines. For example, cell death of the A549 cell line occurred at concentrations as low as 10 ppm, and its cell viability decreased further at 20 and 100 ppm. All measured cells showed a Te‐concentration‐dependent cell viability. Thus, the neat Te in the nanowire form appears toxic (Figure 4f). However, all Te–Se‐based nanomaterials including Te–Se (1/0.25), Te–Se (1/0.5), Te–Se (1/0.75), and Te–Se (1/1) exhibited nontoxic characteristics, even at the highest concentration of 400 ppm (Figure 4a–c,f). During the in vitro experiments, the neat Te nanowires degraded rapidly in saline solution, whereas the Te–Se‐based nanomaterials remained stable at least for 30 days in air (data not shown here). Thus, we speculate that the Te toxicity derives from its degradation products, such as TeO_3_^2−^ (Figure 4f).

**Figure 4:**
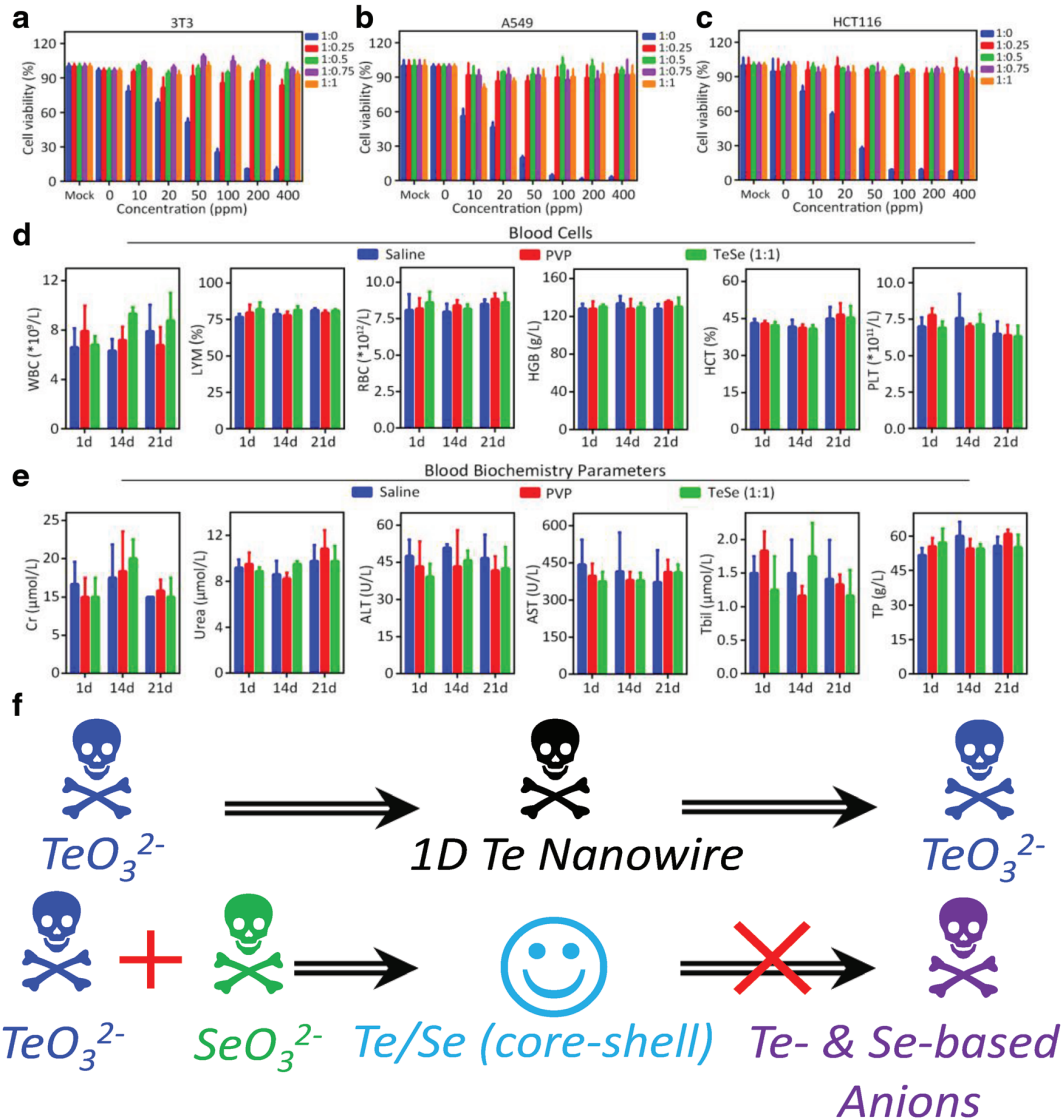
Biocompatibility and biosafety assessment of neat Te wires and Te–Se‐based nanomaterials. Assessment of cell viabilities of a) 3T3, b) A549, and c) HCT116 cells; blood analysis of mice (C57BL/6) intravenously injected with saline, vehicle (PVP solution), or Te–Se (1/1) with a volume of 100 µL at various stages (1, 14, and 21 days). Injection dosage of Te–Se (1/1) is 5 mg kg^−1^. d) Complete blood cells count and e) biochemistry measurements for the indicated parameters. The WBC, LYM, RBC, HGB, HCT, and PLT denote whole blood cell, lymphocyte, red blood cell, hemoglobin, hematocrit, and platelet, respectively. For the biochemical indexes, Cr, ALT, AST, Tbil, and TP denote creatinine, alanine aminotransferase, aspartate aminotransferase, total bilirubin, and total protein, respectively. f) Toxicity or biosafety of Te–Se‐based nanomaterials, Te‐based nanowires, and their precursors.

We also assessed the effects of Te–Se (1/1) nanomaterials on the blood cell count and biochemistry parameters at different times when injected into mice. We examined the whole blood cell number (WBC), number (LYM) and percentage of lymphocytes [LYM (%)], and the number and key characteristics of the red blood cells, namely, hemoglobin (HGB), hematocrit (HCT), and platelet (PLT) characteristics as shown in Figure 4d. The results of the Te–Se (1/1) group showed no differences from those of the respective negative controls of the saline solution and PVP. The levels of creatinine (Cr), urea, alanine aminotransferase (ALT), aspartate aminotransferase (AST), total bilirubin (Tbil), and total protein (TP), which indicate kidney and liver function, remained normal after Te–Se (1/1) administration. Thus, we confirmed that Te–Se (1/1) was not harmful to the mice at 1, 14, and 21 days, as shown in Figure 4e. Therefore, we demonstrated that the Se coating stabilized the Te nanomaterials, and the resulting Te–Se‐based nanomaterials have an extremely low toxicity and a good biocompatibility and biosafety (Figure 4f).

We introduced the Te–Se nanomaterials onto a commercially available and inexpensive MS. The 3D skeleton of the MS (**Figure** **5**a) acts as a support to retain the Te–Se nanomaterials and enable multiple usage cycles. This structure provides a continuously supply of water for solar evaporation owing to its large pore network structure. We first pretreated the MS surface, with a positively charged polyelectrolyte, PDDA, to ensure good adhesion of the Te–Se nanomaterials (Figure 5b). The PDDA@MS was coated with Te–Se nanomaterials to form Te–Se@PDDA@MS (Figure 5c). The π–π interaction and hydrogen bonding of MS with the PDDA chains likely promoted the adhesion of PDDA to MS (Figure S1, Supporting Information), which resulted in positively charged MS (Figure 5d,e). The tails of the PVP chains interacted physically with the MS by hydrogen‐bonding interactions (Figure S1, Supporting Information) and a possible electrostatic interaction occurred between the positively charged PDDA chains and negatively charged Te–Se nanomaterials (Table S2, Supporting Information). In this way, the PDDA@MS can be coated strongly with the Te–Se nanomaterials (Figure 5f) and they are extraordinarily stable in water at room temperature and even at 100 °C without any apparent falling off for the Te–Se nanomaterials (Figure S2, Supporting Information). We investigated the effects of Te–Se (1:1) loading level on the surface morphologies of the MS, as shown in Figure 5g–r. The PDDA‐modified MS had typical 3D networks with many large connected open pores with a wide size range of 85–170 µm (Figure 5g), which is similar to neat MS without PDDA (Figure S3, Supporting Information). We observed clean and smooth surfaces on the PDDA‐treated skeletons (Figure 5h). The Te–Se‐coated PDDA@MS showed several different morphological features at different loading levels. For Te–Se‐coated PDDA@MS at a Te–Se loading of 39.3 wt%, some pores became plugged with Te–Se‐induced thin films (i.e., PVP–Te–Se film) (Figure 5i,j). Moreover, adhesion of Te–Se nanomaterials was observed onto the surfaces of skeleton (Figure 5j). High‐magnification images of the skeleton surface (Figure 5k) showed the homogeneous accumulation of Te–Se nanomaterials. The formed Te–Se‐based films also showed a nanomaterial‐accumulated morphology (Figure 5l). In cross‐sectional images of the Te–Se‐coated PDDA@MS, we did not observe the Te–Se‐induced film (Figure 5m); however, the skeletons were coated by Te–Se nanomaterials, as shown in Figure 5n,o. Furthermore, as the loading of the Te–Se nanomaterials increased, the Te–Se‐induced film thickness on the skeleton surfaces increased gradually [74.1 wt% (Figure 5p), 103.4 wt% (Figure 5q), and 133.4 wt% (Figure 5r)]. The successful loading of Te–Se nanomaterials onto a 3D skeleton suggests the validity of our strategy, which we attribute to the following two reasons: 1) the PVP tails on the surface of the Te–Se nanomaterials were entangled with the MS skeletons and 2) the presence of positively charged PDDA chains on the skeletons interacted with the negatively charged Te–Se nanomaterials through electrostatic interactions.

**Figure 5:**
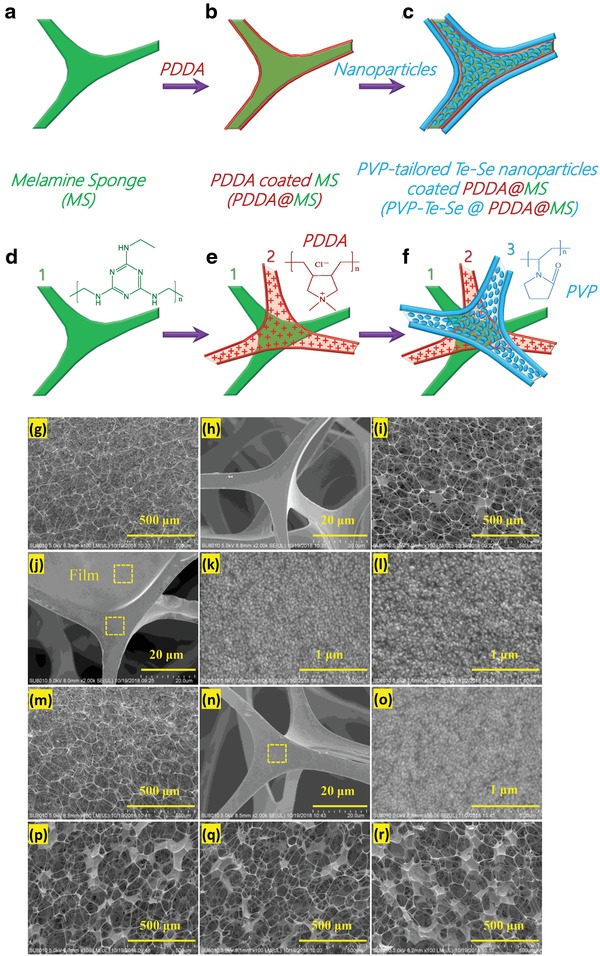
Schematic illustration of the fabrication of Te–Se nanomaterial–decorated MS (Te–Se@PDDA@MS): a,d) neat MS with 3D skeletons; b,e) positively charged PDDA‐modified MS (PDDA@MS); c,f) coating of Te–Se nanomaterials onto PDDA‐pretreated MS. SEM images of g,h) MS‐based samples of PDDA‐modified MS, and Te–Se (1:1)@PDDA@MS with loading levels of Te–Se nanomaterials: i–o) 39.3 wt%, p) 74.1 wt%, q) 103.4 wt%, and r) 133.4 wt%. Note that the images (i–l, p–r) and (m–o) show surface morphologies and cross‐section morphologies of the MS‐based samples, respectively.

A bionic solar‐evaporation system was built to evaluate the solar‐evaporation behavior of Te–Se@PDDA@MS, as shown in **Figure** **6**a, where the Te–Se@PDDA@MS sample worked as a photothermal conversion layer and a white hydrophilic sponge as a water supplement pump because of its strong capillary force. Our photothermal conversion system has several advantages: 1) isolating the photothermal materials from bulk water can reduce energy losses and ensure localized heating and thus an excellent heat utilization; 2) the capillary force of the hydrophilic sponge can supply the photothermal materials with water continuously; and 3) a balanced rate of water evaporation and water supply can be achieved readily. At 1 sun, and relative to an initial temperature of 27.3 °C (Figure 6b), the Te–Se@PDDA@MS sample (74.1% of Te–Se loading) displayed increased temperatures of 39.1 (Figure 6c) and 38.9 °C (Figure 6d) after a respective irradiation time of 375 and 1800 s. We also observed the hydrophilic sponge and bulk NaCl solution (3.5 wt%) with no significant changes in temperature, which indicated a very low heat loss of this bionic solar‐evaporation system, as shown in Figure 6c,d. No salting‐out phenomenon was observed after measurement (Figure 6e), which is of great importance for solar seawater treatment because the crystallized salt from seawater can reflect solar light, which reduces the photothermal efficiency of the materials. During light illumination, the Te–Se@PDDA@MS sample can reach a relatively stable temperature of ≈40°C, which implies a favorable photothermal stability (Figure 6f). A further cycle assessment of the evaporation behaviors of a Te–Se@PDDA@MS sample with a loading level of 74.1 wt% was conducted under 1 sun in NaCl solution, as shown in Figure 6g. An average evaporation efficiency (η) of 86.14 ± 1.02% and evaporation rates (*R*) of 1.323 ± 0.015 kg m^−2^ h^−2^ were calculated, which suggests the excellent evaporation behavior of our samples even under 1 sun relative to other typical materials in the literature (Table S3, Supporting Information).

**Figure 6:**
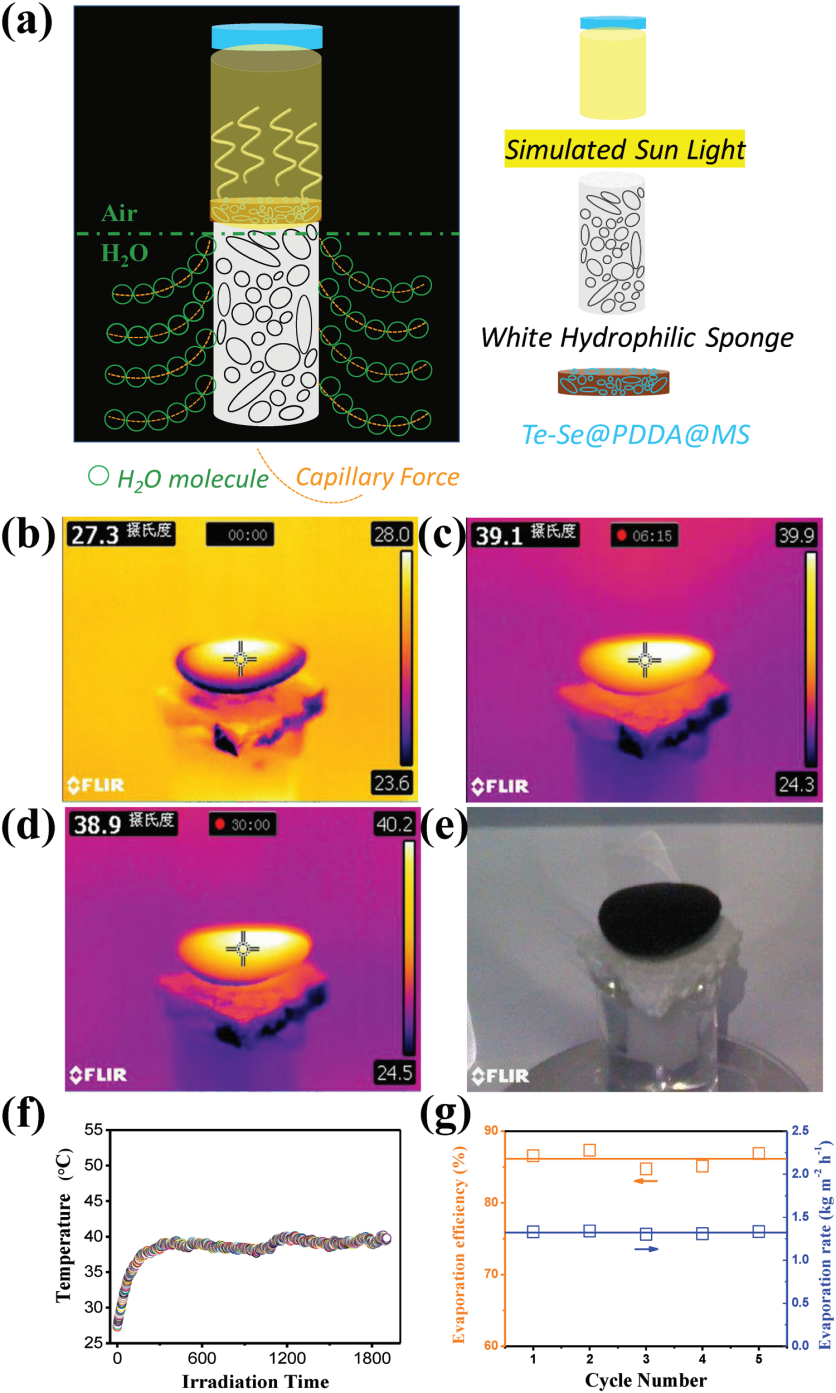
Vapor generation of Te–Se@PDDA@MS with a loading of 74.1 wt% of Te–Se nanomaterials under 1 sun (1 sun = 1 kW m^−2^) for NaCl solution (3.5 wt%). a) A schematic illustrating a bionic solar‐evaporation system. b–d) Infrared images showing the temperature distribution of the whole system after irradiation times of 0, 375, and 1800 s. e) The photograph of Te–Se@PDDA@MS sample after desalination. f) Light‐induced temperature as a function of irradiation time. g) Calculated evaporation efficiency as well as rate for the Te–Se@PDDA@MS sample in each cycle under 1 sun.

The effects of Te–Se (1:1) nanomaterial loading level on the evaporation behaviors of deionized (DI) water, NaCl solution (3.5 wt%), and MgCl_2_ solution (3.5 wt%) were also evaluated under 10 sun in **Figure** **7**. For DI water evaporation under 10 sun irradiation (Figure 7a), the water mass change can be increased by increasing the content of Te–Se from 39.3 to 74.1 wt%. However, the loading level of the Te–Se nanomaterials had little influence on the water mass change from 74.1 to 103.4 wt% even up to 133.4 wt%. Similar results were obtained for NaCl (Figure 7b) and MgCl_2_ (Figure 7c) solutions. We noted a slight effect of the loading level of Te–Se nanomaterials on the water mass change in the MgCl_2_ solution (Figure 7c) when comparing sample 2 (75.5 wt%) with sample 4 (140.7 wt%). According to the scanning electron microscope (SEM) images in Figure 5i–l, a loading level of 39.3 or 41.0 wt% of Te–Se nanomaterials coated the 3D skeletons of the MS adequately. A higher loading of Te–Se nanomaterials may cause films to form over the pores, which reduces the evaporation behavior slightly. The calculated η and *R* values of all measured samples are shown in Figure 7d and Table S4 (Supporting Information). For DI water, a highest η value of 93.39 ± 0.49 was found for sample 2 with a 74.1 wt% loading level of Te–Se nanomaterials, and a corresponding *R* value as high as 13.26 ± 0.069 kg m^−2^ h^−1^ was observed, as shown in Table S4 (Supporting Information). The loading level of the Te–Se nanomaterials onto MS did not have a notable effect on the η or *R* values. However, the evaporation efficiencies and rates for the DI water samples were higher than those of the salt solutions (NaCl and MgCl_2_); evaporation from NaCl solution was slightly better than that from MgCl_2_ (Figure 7d; Table S4, Supporting Information). We did not observe salting out for NaCl and MgCl_2_ solutions even under 10 sun illumination for 3 h (Figure 7e), which suggests an excellent decontamination salt‐resistance performance. It is considered that there exists a temporary concentration gradient between the solution near the surface of Te–Se@PDDA@MS sponge and bulk solution far away from the sponge under the evaporation process. However, the excellent water absorption capability of our sponge, originated from its multipore structure, can continuously supplement fresh solution from bulk solution for evaporation, and the big enough 3D space within pores also can cause a readily convection between the evaporation water layer and the bulk water layer. As a result, the salt concentration near the sponge samples will always be lower than the required saturated concentration of crystallization. This can be well responsible for the excellent antifouling performance of our Te–Se@PDDA@MS sponges. Absorption spectra of all solid samples wetted with water or NaCl solution are shown in Figure 7f. Neat MS or PDDA‐modified MS showed ≈50–60% absorption from 300 to 2500 nm, which may be attributed to multiple reflections inside the pores, which scattered and trapped incoming light.^31^, ^32^ After Te–Se nanomaterial coating, we observed ≈93–95% light absorption. Some samples that were wetted by NaCl solution showed a slightly decreased absorption, but the overall absorption exceeded 93%. The above absorption results suggest that the excellent evaporation properties of the Te–Se@PDDA@MS samples originated from the Te–Se photothermal materials. The slightly lower absorption of the samples wetted with NaCl solution likely contributes to the slightly lower η and *R* values compared with those features of the DI water samples.

**Figure 7:**
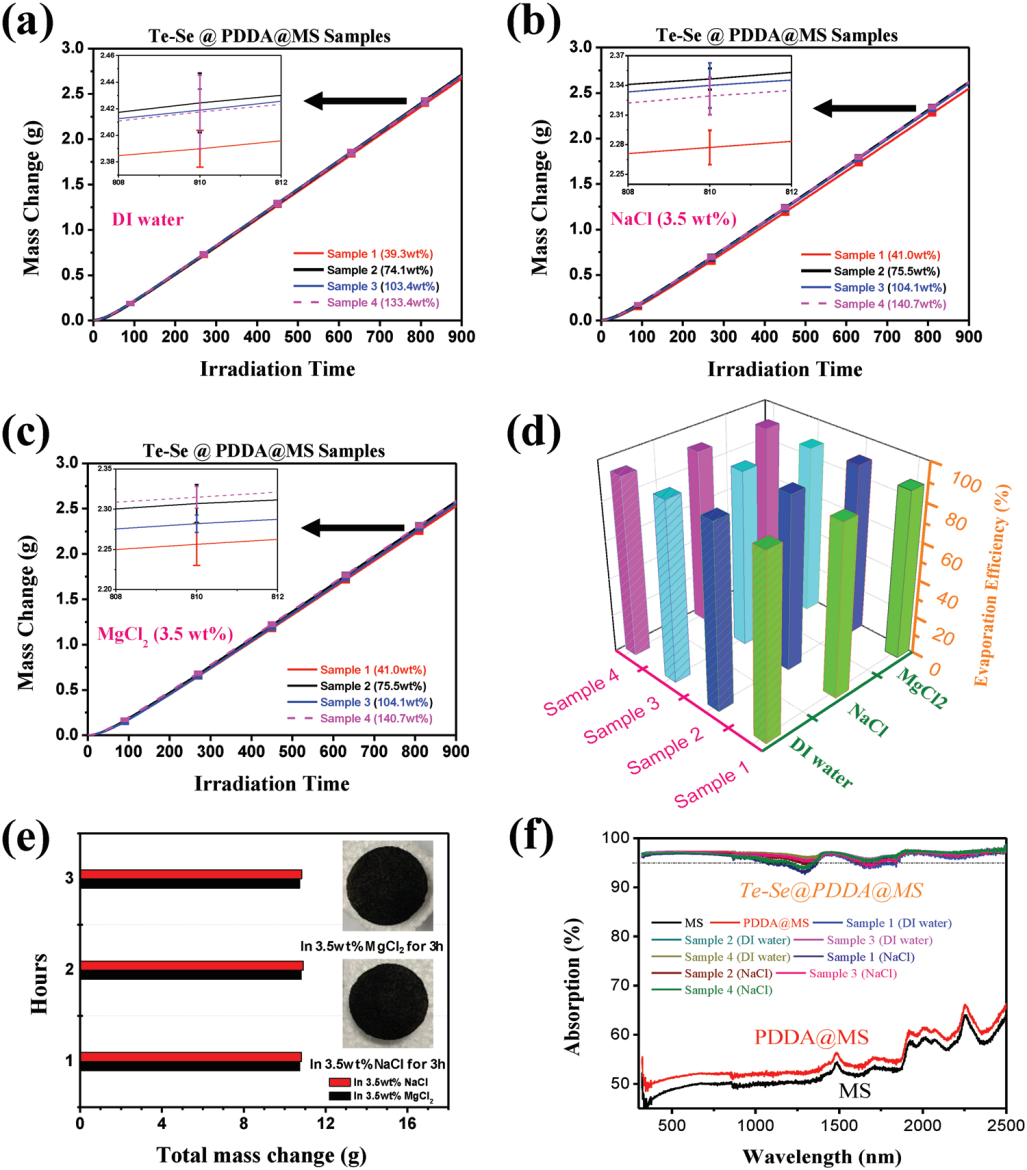
Vapor generation of Te–Se@PDDA@MS under 10 sun. Water mass loss as a function of solar irradiation time for a) DI water, b) 3.5 wt% NaCl solution, and c) 3.5 wt% MgCl_2_ solution under 10 sun. d) Corresponding evaporation efficiency (η) of various samples. e) Salt‐resistant behaviors of sample 2 for a long vapor‐generation time of 3 h (inset: photographs of samples after 3 h). f) Absorption spectra of MS‐based samples wetted with DI water and NaCl solution (3.5 wt%) for 300–2500 nm.

Integrated vapor generation and water‐collection equipment was built based on quartz units (**Figure** **8**a). During vapor generation, the water vapor can be condensed onto the quartz surface (Figure 8b), and be collected for measurement. In this study, a real seawater specimen (Shenzhen Bay, Shenzhen, China) was used for solar desalination via the Te–Se@PDDA@MS sample with a 74.1 wt% loading level of Te–Se nanomaterials under natural light. After an entire day of vapor generation, the Te–Se@PDDA@MS sample did not show any salting‐out phenomenon, as shown in the inset photograph in Figure 8b, which suggests its salt‐resistant performance. The detected ion concentration in the collected water was 4 ppm, by using a commercial portable MIUI‐type electronic product (Figure 8c). The concentrations of four primary ions (Na^+^, Mg^2+^, K^+^, and Ca^2+^) in the seawater and the collected water are shown in Figure 8d. Four ion types had significantly reduced concentrations after desalination and were far below 103 mg L^−1^ as suggested by the World Health Organization.^33^

**Figure 8:**
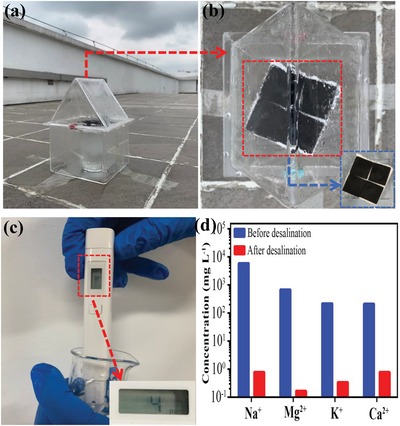
Integrated vapor generation and water‐collection equipment for real seawater under natural light. a) Quartz‐based units for building equipment. b) Corresponding top view that can observe condensate water. Inset: photograph of Te–Se@PDDA@MS sample after a 1‐day vapor‐generation experiment. c) Detection of ion concentration of collected water by using commercial MIUI detector. d) Ion concentrations of real seawater and corresponding collected fresh water by desalination under natural light.

We developed novel 2D core–shell Te–Se nanomaterials with excellent photothermal properties and biocompatibility and used them to coat 3D skeletons of a commercial MS to develop 3D porous photothermal conversion materials for high‐performance solar desalination. Compared with the toxic 1D nanowires of neat Te, the Te–Se nanomaterials had 2D oval‐like morphologies with a good biocompatibility in vitro and in vivo. The Se coating on the Te altered the Te–Se nanomaterial morphology and stabilized the Te, which likely contributed to the excellent biocompatibility of the Te–Se nanomaterials by preventing toxic Te degradation products from forming. We used a simple approach to integrate the Te–Se nanomaterials into MS to fabricate 3D porous photothermal materials, which, together with another hydrophilic sponge, were built into a bionic solar‐evaporation generation system. A high energy efficiency of ≈86.14 ± 1.02% and a high evaporation rate of 1.323 ± 0.015 kg m^−2^ h^−2^ for Te–Se‐coated MS in NaCl solution at 1 sun were achieved. The energy efficiency was increased to 90.71 ± 0.37% and the evaporation rate was increased to 12.88 ± 0.052 kg m^−2^ h^−2^ in NaCl solution at 10 sun. No salting‐out phenomenon was observed for the NaCl and MgCl_2_ solutions even with long‐term usage, which suggests the viability of our approach. We attribute the high‐performance of this solar desalination to the efficient photothermal material and its arrangement, which separated the photothermal layers from bulk water in the porous structures of the 3D support. This structure prevented salting out, and ensured robust adhesion of the nanosized semiconductors to provide a high photothermal conversion efficiency and excellent biocompatibility. Our work paves the way for the development of 2D Te‐based photothermal materials for solar desalination and wastewater treatment, and is attractive for photothermal therapy in several future biomedical applications.

## Experimental Section

*Chemicals and Materials*: All chemicals and materials were purchased and used without purification, and included sodium tellurite (Na_2_TeO_3_, 97%, Macklin), sodium selenite (Na_2_SeO_3_, ≥98%, Sigma‐Aldrich), PVP (MW = 360 000, Sigma‐Aldrich), ammonium hydroxide solution (NH_3_·H_2_O, 25–28%, Macklin), and hydrazine monohydrate (N_2_H_4_·H_2_O, >98.0%, Aladdin). Bulk crystal powders of tellurium (Te, 99.9%) and selenium (Se, 99.9%), and PDDA solution (20 wt%) were from Aladdin Company. Commercial melamine sponge was from the Building Materials of Beiyou Company, Shanghai, China. All solvents were of analytical reagent grade.

*Synthesis of Te–Se Nanomaterials*: A common hydrothermal reaction strategy was used to produce Te‐based nanomaterials with or without Se doping. In brief, the Te source (Na_2_TeO_3_), Se source (Na_2_SeO_3_), and a PVP surfactant were dissolved in DI water. Then, a certain volume of N_2_H_4_·H_2_O (reducing agent) and NH_3_·H_2_O were added to the above homogeneous solution. After stirring for 5 min, the mixture was added to a Teflon‐lined stainless steel autoclave for a hydrothermal reaction at 100 °C for 3 h. After reaction, the cooled product solution was dialyzed with DI water by using dialysis bags (MWCO: 3500 Da) for a few days until the pH was 7. The complete dialysis process is of significance for evaluating the biocompatibility and biosafety of Te–Se samples from the perspective of the toxicity of the Te source, Se source, NH_3_·H_2_O, and N_2_H_4_·H_2_O. A freeze‐drying procedure was used to obtain solid Te–Se nanomaterials. Various initial molar ratio values of Te to Se source (such as 1/0.25, 1/0.5, 1/0.75, and 1/1) were used to produce Te–Se nanomaterials. The PVP solution concentration was 2 mg mL^−1^ (mass/H_2_O). The synthesis information of all samples is listed in detail in Table S5 (Supporting Information). For comparison, the Te‐only sample [(Te–Se (1/0)] was also prepared, according to the similar method above without addition of a Se source.

*PDDA‐Modified MS*: The MS was cut into small circle‐shaped pieces with a diameter of ≈33 mm and a thickness of 1 mm and cleaned ultrasonically in acetone, ethanol, and DI water for 30 min, followed by dying at 85 °C. The positively charged polyelectrolyte, PDDA, was used to modify MS. Briefly, the cleaned and weighed MS was dipped into an aqueous 2 wt% PDDA solution at a slow agitation speed for 12 h. The PDDA‐soaked MS was squeezed to remove excess solution and then dipped into the PDDA solution again. This step was repeated several times to wet the MS thoroughly. Finally, the as‐prepared MS samples were squeezed to remove the PDDA solution, dried, and dipped into hot water at 70 °C at a slow agitation speed to remove weakly absorbed PDDA chains. The obtained PDDA@MS samples were dried at 85 °C and their masses were recorded.

*Preparation of Te–Se@PDDA@MS*: The preparation of Te–Se@PDDA@MS samples was done as follows: 1) the PDDA@MS samples were dipped into hot water to wet the surface (dried PDDA@MS was hydrophobic owing to the presence of PDDA on the surfaces of the hydrophilic MS) and then fully wetted with 4 mL of Te–Se (1/1) solution; 2) the MS‐based sample was dried with a low‐power electric hair drier for at least 10 min and then dried at high power before being transferred to an air‐drying oven at 85 °C; 3) the dried MS samples were dipped into water and squeezed and pressed several times, to remove weakly absorbed Te–Se nanomaterials; and 4) the above three steps were repeated to achieve a greater absorption of Te–Se nanomaterials on the PDDA@MS. The contents of Te–Se nanomaterials were determined from the mass change.

*Biocompatibility and Biosafety Assessment—Cell Culture*: Mouse embryonic fibroblast 3T3 cell line, human A549 lung carcinoma epithelial cells, and human colorectal carcinoma cell line HCT116 were from ATCC. Cells were cultured in Dulbecco's modified Eagle medium (Hyclone) with 10% fetal bovine serum (Hyclone), 100 U/mL penicillin, and 100 µg/mL streptomycin (Hyclone).

*Biocompatibility and Biosafety Assessment—Animals*: All animal studies were approved by the Animal Ethics Committee of Shenzhen University. Female C57BL/6 and nude BALB/c mice (4–6 weeks old) were from the Guangdong Medical Laboratory Animal Center in Guangdong, China. Mice were maintained under standard conditions.

*Biocompatibility and Biosafety Assessment—In Vitro Toxicity*: For the in vitro toxicity studies, 8000 cells per well were inoculated in 96‐well plates. After adherence, the medium was replaced with a nanoparticle‐containing medium and cells were cultured for 36 h before CCK8 assays (Beyotime Technology, Shanghai, China). Cell viabilities were normalized to negative controls.

*Biocompatibility and Biosafety Assessment—In Vivo Toxicity*: To determine the in vivo toxicity, the Te–Se (1/1) nanomaterial was injected intravenously into C57BL/6 mice. At different times, the mice were sacrificed and their blood was collected for complete blood counts and blood chemistry measurements. The body masses of each mouse were monitored at 2‐day intervals.

*Evaporation Experiments*: The above Te–Se@PDDA@MS samples with a diameter of ≈33 mm and a thickness of 1 mm were used for solar steam‐generation tests. The sample evaporator was laid on a 25 mL beaker filled with DI water, NaCl solution (3.5 wt%), or MgCl_2_ solution (3.5 wt%). An AM 1.5 light filter, two xenon lamps with modes of CEL‐HXF300 (Aulight Co. Ltd.), and a Solar‐1000 (NBeT Co. Ltd.) that was adjusted to an aperture of 33 mm were used to simulate sunlight with a power density of 10 kW m^−2^ (10 sun) and 1 kW m^−2^ (1 sun). The optical power density was measured by using an optical power meter (CEL‐NP2000, Aulight Co. Ltd.). Electronic scales (QUINTIX124‐1CN and Practum213‐1CN, Sartorius), which can be connected to a computer, were used to record the mass change with time. The solar‐evaporation efficiency (η) was calculated from

(1) $$\eta \;=\frac{\dot{m}h_{\mathrm{LV}}}{C_{\mathrm{opt}\;}q_{i}}$$

(2) $$\;h_{\mathrm{LV}}=\;Q+L_{v}$$

(3) $$L_{v}=\;1.91846\times 10^{3}{\left[T_{i}/\left(T_{i}-33.91\right)\right]}^{2}\;J\;g^{-1}$$

(4) $$Q=c\left(T_{i}-T_{s}\right)\;J\;g^{-1},\;\;c=4.2\;J\;g^{-1}\;K^{-1}$$

where $\dot{m}$, *h*_LV_, *C*_opt_, and *q*_i_ denote the mass flux (i.e., evaporation rate), total enthalpy of the gas–liquid phase change (enthalpy of phase change and sensible heat), optical concentration on the absorber surface, and the normal solar radiation intensity (1 kW m^−2^), and Ts and Ti denote the temperatures of the bulk water and its evaporation surface under illumination, respectively. All calculations were made by deducting natural evaporation data in the dark. The temperature change during a 30 min evaporation under 1 sun illumination in a laboratory environment (temperature of 21–24 °C and humidity of 45–55%) was measured by using an infrared thermal imaging camera (FLIR‐160).

A real seawater sample from Shenzhen Bay (Shenzhen, China) was used for solar desalination via a Te–Se@PDDA@MS evaporator under natural sunlight (time: November 12–14, 2018; temperature: 20–25 °C; location: Xili Campus of Shenzhen University, Shenzhen, China). The collected fresh water and the original seawater were analyzed to determine their ion concentrations (including Na^+^, K^+^, Ca^2+^, and Mg^2+^) by using ICP‐AES (OPTIMA2100DV, PerkinElmer).

*Modeling Methods*: The simulations in this study were conducted by using first‐principles density functional theory (DFT). The ground‐state wave functions were calculated by using the Perdew–Burke–Ernzerhof exchange–correlation functional^34^ in the Quantum Espresso code^35^ with scalar‐relativistic norm‐conserving pseudopotentials.^36^ A 60 Ry kinetic energy cutoff and 1 × 1 × 4 *k*‐point sampling were applied and the energy convergence was 10^−5^ eV per atom.

*Characterization*: Morphological images of the as‐prepared Te‐only and Te–Se nanomaterials were obtained by using TEM (Tecnai G2 Spirit). High‐resolution TEM (HRTEM) was also performed to detect the crystal lattice and element distribution of nanoparticles by using a double spherical aberration‐corrected transmission electron microscope. AFM (Bruker) was used to determine the thickness of the Te–Se samples with a tapping mode. X‐ray photoelectron spectroscopy (XPS) [PHI‐5000 VersaProbe II (ULVAC‐PHI) instrument] with monochromatic Al Kα radiation was performed to evaluate the valence states of the elemental Te and to detect the dialysis efficiency of the samples. ICP‐AES was used to determine the mass content of the Te phase, Se phase, and PVP of the Te–Se nanomaterials. The crystalline characteristics of the bulk Te, bulk Se, and Te–Se samples were tested by XRD and Raman measurements (Horiba LabRAM HR800). The XRD patterns were recorded with a scanning speed of 2° min^−1^ from 20° to 70° at room temperature. The Raman measurements were excited at 633 nm at room temperature. The optical absorption of the Te–Se samples was measured by using a UV–vis absorbance spectrometer (Cary 60, Agilent) from 200 to 1200 nm. The optical absorption of Te–Se‐coated PDDA@MS was detected in a wet state. The reported absorption values were obtained by deducting the reflection and transmittance contributions. The surface and cross‐sectional morphologies of the neat MS, PDDA@MS, and Te–Se‐coated PDDA@MS were observed by SEM (Hitachi‐SU8010). Owing to their excellent flexibility, cross‐sections of the MS samples were obtained by cutting rather than fracturing after quenching in liquid nitrogen. The samples were coated with a thin layer of silver before characterization. An acceleration voltage of 5 kV was used.

## Conflict of Interest

The authors declare no conflict of interest.

## Supporting information

Supplementary
