# Supplementary material for: Engineering Lateral Heterojunction of Selenium‐Coated Tellurium Nanomaterials toward Highly Efficient Solar Desalination
Source: Adv Sci (Weinh). 2019 Aug 17;6(19):1900531. doi: 10.1002/advs.201900531 (PMC6774058; doi:10.1002/advs.201900531)
Supplement: Supplementary file 1 — Supplementary [file ADVS-6-1900531-s001.pdf]

Copyright WILEY-VCH Verlag GmbH & Co. KGaA, 69469 Weinheim, Germany, 2019.

ADVANCED SCIENCE
Open Access

Supporting Information

for *Adv. Sci.,* DOI: 10.1002/advs.201900531

Engineering Lateral Heterojunction of Selenium-Coated Tellurium Nanomaterials toward Highly Efficient Solar Desalination

Chenyang Xing, Dazhou Huang, Shiyou Chen, Qichen Huang, Chuanhong Zhou, Zhengchun Peng, Jiagen Li, Xi Zhu, Yizhen Liu,* Zhipeng Liu, Houkai Chen, Jinlai Zhao, Jiangqing Li, Liping Liu, Faliang Cheng, Dianyuan Fan, and Han Zhang**

Supporting Information

Engineering Lateral Heterojunction of Selenium-Coated Tellurium Nanomaterials Towards Highly Efficient Solar Desalination

Chenyang Xing,[⊥ab] Dazhou Huang, [⊥ai] Shiyou Chen, [⊥a] Qichen Huang, [⊥d] ,Chuanhong Zhou,[b] Zhengchun Peng,[b] Jiagen Li,[c] Xi Zhu,[c]* Yizhen Liu,[d]*, Zhipeng Liu, [d] Houkai Chen,[e] Jinlai Zhao,[fg] Jiangqing Li,[f] Liping Liu,[h] Faliang Cheng,[i] Dianyuan Fan,[a] Han Zhang[a]*

[a] Shenzhen Engineering Laboratory of Phosphorene and Optoelectronics, International Collaborative Laboratory of 2D Materials for Optoelectronics Science and Technology of Ministry of Education, College of Physics and Optoelectronic Engineering, Shenzhen University, Shenzhen 518060, China

[b] Center for Stretchable Electronics and Nanoscale Systems, Key Laboratory of Optoelectronic Devices and Systems of Ministry of Education, College of Physics and Optoelectronic Engineering, Shenzhen University, Shenzhen 518060, China

[c] School of Science and Engineering, The Chinese University of Hong Kong, Shenzhen, Shenzhen 518172, China

[d] College of Chemistry and Environmental Engineering, Shenzhen University, Shenzhen 518060, China

[e] Nanophotonics Research Center, Shenzhen Key Laboratory of Micro-Scale Optical Information Technology, Shenzhen University, Shenzhen 518060, China

[f] Faculty of Information Technology Macau University of Science and Technology, Avenida Wai Long, Taipa, Macau 999078, China

[g] College of Materials Science and Engineering, Shenzhen Key Laboratory of Polymer Science and Technology, Guangdong Research Center for Interfacial Engineering of Functional Materials, Shenzhen 518060, China

[h] Department of Hepatobiliary and Pancreatic Surgery, Shenzhen People’s Hospital, Second Clinical Medical College of Jinan University, Shenzhen 518060, China

[i] Dongguan University of Technology, Dongguan 523808, China

┴ C. Y. Xing, D. Z. Huang, S. Y. Chen and Q. C. Huang contributed equally.

[*] Corresponding author (E-mail: hzhang@szu.edu.cn; yzliu@szu.edu.cn; zhuxi@cuhk.edu.cn)

Contents

Table S1 Weight percent values of Te phase, Se phase, and PVP component in the Te-Se-based nanomaterials, determined by inductively coupled plasma-atomic emission spectroscopy (ICP-AES, OPTIMA2100DV, PerkinElmer).

Table S2 The zeta potential of DI water, PVP solution and Te-Se (1/1) solution at 25 °C.

Table S3 Comparison of Te-Se@PDDA@MS vapor generation performance and previous reports. The *η*, *R*, NPs, GBMCC, CNTs denote evaporation efficiency, evaporation rate, nanoparticles, geopolymer-biomass mesoporous carbon composite and carbon nanotubes, respectively. The symbol of "–" means this kind of data had not been mentioned in the literatures.

Table S4 Evaporation efficiency (*η*, %), evaporation rate (*R*, $\mathrm{kg}\square\mathrm{m}^{-2}\square\mathrm{h}^{-1}$) of Te-Se (1/1) @PDDA@MS samples with various loading level (W, wt%) of Te-Se (1/1) nanomaterials for DI water, NaCl solution (3.5 wt%), and $MgCl_2$ solution (3.5 wt%) under a solar intensity of 10 sun (1 sun = 1 $\mathrm{kW}\square\mathrm{m}^{-2}$). Weight changes ($\Delta$W, wt%) of the MS samples before and after measurements are also shown.

Table S5 The synthesis information of all samples in this study.

Fig. S1 Chemical structures of PDDA (a, b), MS (c) and PVP, and their ATR-FTIR spectra relative to Te-Se (1/1) decorated PDDA@MS sample.

Fig. S2 (a) Photographs of neat MS, PDDA@MS, Te-Se@PDDA@MS with Te-Se loadings of 39.3 wt%, 74.1 wt%, 103.4 wt% and 133.4 wt%, respectively (from left to right); The free states (b) and bending states (c) of Te-Se@PDDA@MS with a Te-Se loading of 133.4 wt% in boiling water and room-temperature water.

Fig. S3 SEM images illustrating the surface morphology of neat MS without any treatment.

Table S1 Weight percent values of Te phase, Se phase, and PVP component in the Te-Se-based nanomaterials, determined by inductively coupled plasma-atomic emission spectroscopy (ICP-AES, OPTIMA2100DV, PerkinElmer).

Sample name	Te Phase (wt%)	Se Phase (wt%)	PVP (wt%)	Molar ratio of Te to Se (Theory)	Molar ratio of Te to Se (Experiments)
Te-Se (1/0.25)	48.2	6.4	45.4	4	4.67
Te-Se (1/0.5)	46.0	11.3	42.7	2	2.52
Te-Se (1/0.75)	43.1	15.6	41.3	1.33	1.71
Te-Se (1/1)	39.6	18.4	42	1	1.33

Table S2 The zeta potential of DI water, PVP solution and Te-Se (1:1) solution at 25 °C.

Materials	Zeta Potential (mV)
Water	8.6 ± 1.8
PVP/Water	4.93 ± 0.88
Te-Se (1/1)/Water	19.2 ± 1.1

Table S3 Comparison of Te-Se@PDDA@MS vapor generation performance and previous reports. The η, R, NPs, GBMCC, CNTs denote evaporation efficiency, evaporation rate, nanoparticles, geopolymer-biomass mesoporous carbon composite and carbon nanotubes, respectively. The symbol of "–" means this kind of data had not been mentioned in the literatures.

**Materials**	Structures	Biocompatible; Degradable	η	R ($kg \cdot m^{-2} \cdot h^{-1}$)	Water Source	Salting-Out	Reference
MXene Ti_3C_2	3D Thin Membrane	Yes; Yes	84% (1sun)	1.33 (1 sun)	Fresh water	−	1
Au NPs	3D Au@ Filter Paper	Yes; NO	79% (0.9 sun) 89% (10 sun)	0.97 (0.9 sun)	Fresh water	Yes	2
Black Au	3D Au@ Sponges	−; NO	90.3% (10 sun)	12.74 (10 sun)	NaCl solution	NO	3
GBMCC	3D Porous Structure	Yes; NO	84.95% (1 sun)	1.58 (1 sun)	NaCl solution	−	4
$CuFeSe_2$ NP	3D NP@Wood Membrance	−; NO	67.7% (1 sun) 86.2% (5 sun)	6.6 (5 sun)	Fresh water	−	5
Graphene	3D foam	−; NO	91.4% (1 sun)	1.4 (1 sun)	Sea Water	−	6
Ti_2O_3 NPs	3D Ti_2O_3@ Cellulose Membrance	−; NO	92.1±3.2 % (1 sun)	1.32 (1 sun) 5.03 (5 sun)	Sea Water	−	7
CNTs	3D CNT@Filter Paper	−; NO	75 % (1 sun)	1.15 (1 sun)	Sea Water	−	8
Polypyrrole	3D Porous hydrogel	−; NO	94 % (1 sun)	3.2 (1 sun)	NaCl solution	−	9
Te NPs	1D nanoparticel	−; NO	−	−	Fresh water	−	10
2D Te-Se	3D Te-Se@Sponges	Yes; NO	93.39±0.49 (10 sun) for fresh water 90.71±0.37 (10 sun) for NaCl (aq) 89.82±0.28 (10 sun) for $MgCl_2$ (aq) 86.14% (1 sun) for NaCl (aq)	13.26±0.069 (10 sun) for fresh water 12.88±0.052 (10 sun) for NaCl (aq) 12.75±0.040 (10 sun) for $MgCl_2$ (aq) 1.323 (1 sun) for NaCl (aq)	NaCl solution	NO	This Work

References:

1. R. Li, L. Zhang and P. Wang, *ACS Nano*, 2017, **4**, 3752.
2. Z. Liu, Z. Yang, X. Huang, C. Xuan, J. Xie, H. Fu, Q. Wu, J. Zhang, X. Zhou and Y. Liu, *J. Mater. Chem. A.*, 2017, **5**, 20044.
3. Y. Liu, Z. Liu, Q. Huang, X. Liang, X. Zhou, H. Fu, Q. Wu, J. Zhang and W. Xie, *J. Mater. Chem. A*, 2018, DOI: 10.1039/C8TA10227A.
4. F. Liu, B. Zhao, W. Wu, H. Yang, Y. Ning, Y. Lai and R. Bradley, *Adv. Funct. Mater.*, 2018, **28**, 1803266.
5. H. Liu, C. Chen, H. Wen, R. Guo, N. A. Williams, B. Wang, F. Chen and L. Hu, *J. Mater. Chem. A*, 2018, **6**, 18839.
6. H. Ren, M. Tang, B. Guan, K. Wang, J. Yang, F. Wang, M. Wang, J. Shan, Z. Chen, D. Wei, H. Peng and Z. Liu, *Adv. Mater.*, 2017, **29**, 1702590.
7. J. Wang, Y. Li, L. Deng, N. Wei, Y. Weng, S. Dong, D. Qi, J. Qiu, X. Chen and T. Wu, *Adv. Mater.*, 2017, **29**, 1603730.
8. P. Yang, K. Liu, Q. Chen, J. Li, J. Duan, G. Xue, Z. Xu, W. Xie and J. Zhou, *Energy Environ. Sci.*, 2017, **10**, 1923.
9. F. Zhao, X. Zhou, Y. Shi, X. Qian, M. Alexander, X. Zhao, S. Mendez, R. Yang, L. Qu and G. Yu, *Nat. Nanotech.*, 2018, **13**, 489.
10. C. Ma, J. Yan, Y. Huang, C. Wang and G. Yang, *Sci. Adv.*, 2018, **4**, eaas9894.

Table S4 Evaporation efficiency (η, %), evaporation rate (R, kg□m^{-2}□h^{-1}) of Te-Se (1/1) @PDDA@MS samples with various loading level (W, wt%) of Te-Se (1/1) nanomaterials for DI water, NaCl solution (3.5 wt%), and $MgCl_2$ solution (3.5 wt%) under a solar intensity of 10 sun (1 sun = 1 kW□m^{-2}). Weight changes (ΔW, wt%) of the MS samples before and after measurements are also shown.

**Samples**	W (wt%)	ΔW (wt %)	η for DI water (%)	R for DI water (kg□m^{-2}□h^{-1})	η for NaCl solution (%)	R for NaCl solution (kg□m^{-2}□h^{-1})	η for $MgCl_2$ solution (%)	R for $MgCl_2$ solution (kg□m^{-2}□h^{-1})
**Sample 1** (DI water)	39.3	< 2.0	92.2±0.42	13.09±0.059	–	–	–	–
**Sample 1** (NaCl or $MgCl_2$)	41.0	< 2.0	–	–	89.22±0.49	12.67±0.069	88.62±0.37	12.59±0.053
**Sample 2** (DI water)	74.1	< 2.0	93.39±0.49	13.26±0.069	–	–	–	–
**Sample 2** (NaCl or $MgCl_2$)	75.5	< 2.0	–	–	90.71±0.37	12.88±0.052	89.82±0.28	12.75±0.040
**Sample 3** (DI water)	103.4.	< 2.0	92.80±0.14	13.17±0.020	–	–	–	–
**Sample 3** (NaCl or $MgCl_2$)	104.1	< 2.0	–	–	90.71±0.48	12.88±0.069	89.82±0.56	12.75±0.079
**Sample 4** (DI water)	133.4	< 2.0	92.79±0.64	13.17±0.091	–	–	–	–
**Sample 4** (NaCl or $MgCl_2$)	140.7	< 2.0	–	–	90.41±0.37	12.84±0.052	89.52±0.24	12.71±0.034

Table S5 The synthesis information of all samples in this study.

Sample names	Mass of Na_2TeO_3 (mg)	Mass of Na_2SeO_3 (mg)	Initial molar ratio: Na_2TeO_3/ Na_2SeO_3	Mass of PVP (mg)	H_2O (mL)	N_2H_4• H_2O (mL)	NH_3• H_2O (mL)
Te-Se (1/0)	221.57	0	1:0	100	50	5	5
Te-Se (1/0.25)	221.57	43.235	1:0.25	100	50	5	5
Te-Se (1/0.5)	221.57	86.47	1:0.5	100	50	5	5
Te-Se (1/0.75)	221.57	129.705	1:0.75	100	50	5	5
Te-Se (1/1)	221.57	172.94	1:1	100	50	5	5

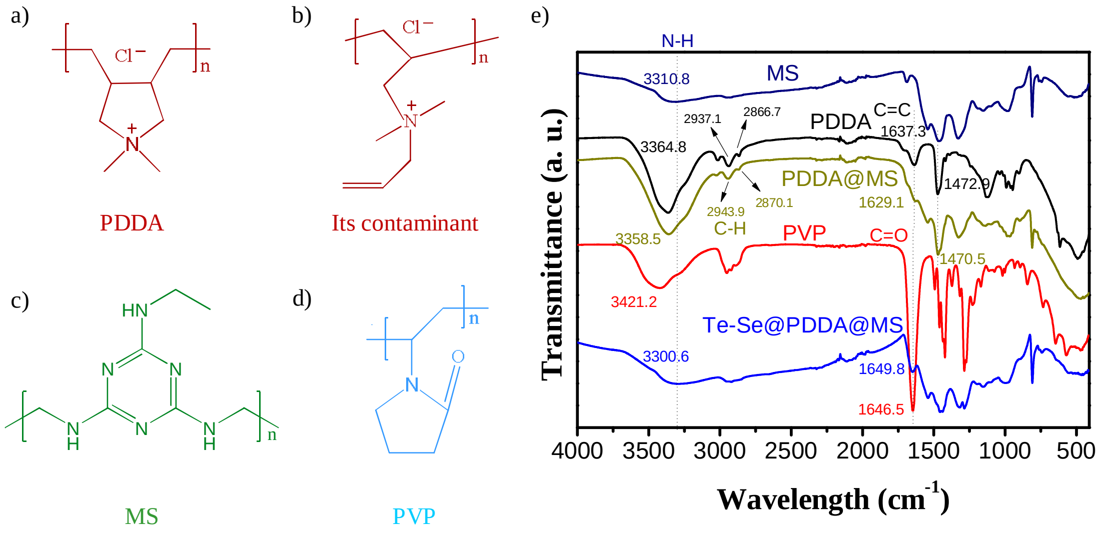

Fig. S1 Chemical structures of PDDA (a, b), MS (c) and PVP, and their ATR-FTIR spectra relative to Te-Se (1/1) decorated PDDA@MS sample.

According to Sacher et al, [1] poly(diallyl dimethylammonium) chloride (PDDA), a positively charged polyelectrolyte, can well interact with carbon nanotubes (CNTs) via a straightforward π-π interaction. And PDDA (**Fig. S1** a) is frequently mixed with its contaminant (**Fig. S1** b) which is formed during the polymerization of the monomer and can be considered as an unsaturated impurity. Its presence can be sufficiently high for orbital overlap, thus permitting π*← π transitions between PDDA and CNTs.

In this work, given its similar C=C formed π-conjugated system to CNTs (**Fig. S1** c), MS was first modified by PDDA. As shown in **Fig. S1 e**, peak at 1637.3 and 1472.9 cm^{-1} of PDDA, assigned to unsaturated C=C bonds of PDDA, [1] shift to low-wavelength of 1629.1 and 1470.5 cm^{-1} in the case of PDDA@MS, the formed of which is accompanied with a significant decrease in intensity. This means a similar π-π interaction between PDDA and MS. It is also observed that peaks at 2937.1 and 2886.7 cm^{-1} from C—H stretching vibration of methylene as well as peaks at 3364.8 cm^{-1} possibly from stretching vibration of O—H of water absorbed onto PDDA in PDDA sample are significantly shifted to higher- and lower-wavelength, respectively,

in the case of PDDA@MS, which may be ascribed to possible hydrogen-bonding interactions between positively charged N^+ of PDDA and H—NH—R of MS. Compared to neat MS at 3310.8 cm^{-1} from stretching vibration of N—H, [2] such a hydrogen-bonding interaction in the PDDA@MS case is also obviously. Therefore, it can be concluded that PDDA can interaction with MS via both π-π interaction and H-bonding interactions.

As for Te-Se @PDDA@MS, the peak at 1649.8 cm^{-1} assigned to C=O (**Fig. S1 d**) from PVP has a about 3 shifts of wavelength compared with that of 1646.5 cm-1 for PVP. Meanwhile, a peak at 3300.6 cm-1 from stretching vibration of N—H in Te-Se @PDDA@MS also has a significant red-shift phenomenon relative to 3358.5 cm^{-1} of neat PVP and 3310.8 cm^{-1} of neat MS, indicating that a possible H-bonding interaction of PVP and MS.

In summary, a robust adhesion of Te-Se nanomaterials onto PDDA@MS can attributed to strong physical interactions, such as π-π interaction (PDDA and MS), H-bonding interactions (PDDA and MS; PVP and MS) and possible electrostatic interaction (positively charged PDDA and negatively charged Te-Se nanomaterials).

References:

(1) D. Yang, J. F. Rochette and E. Sacher, *J. Phys. Chem. B* 2005, **109**, 4481-4484.

(2) W. Zhang, X. Zhai, T. Xiang, M. Zhou, D. Zang, Z. Gao and C. Wang, *J. Mater. Sci.*, 2017, **52**, 73–85.

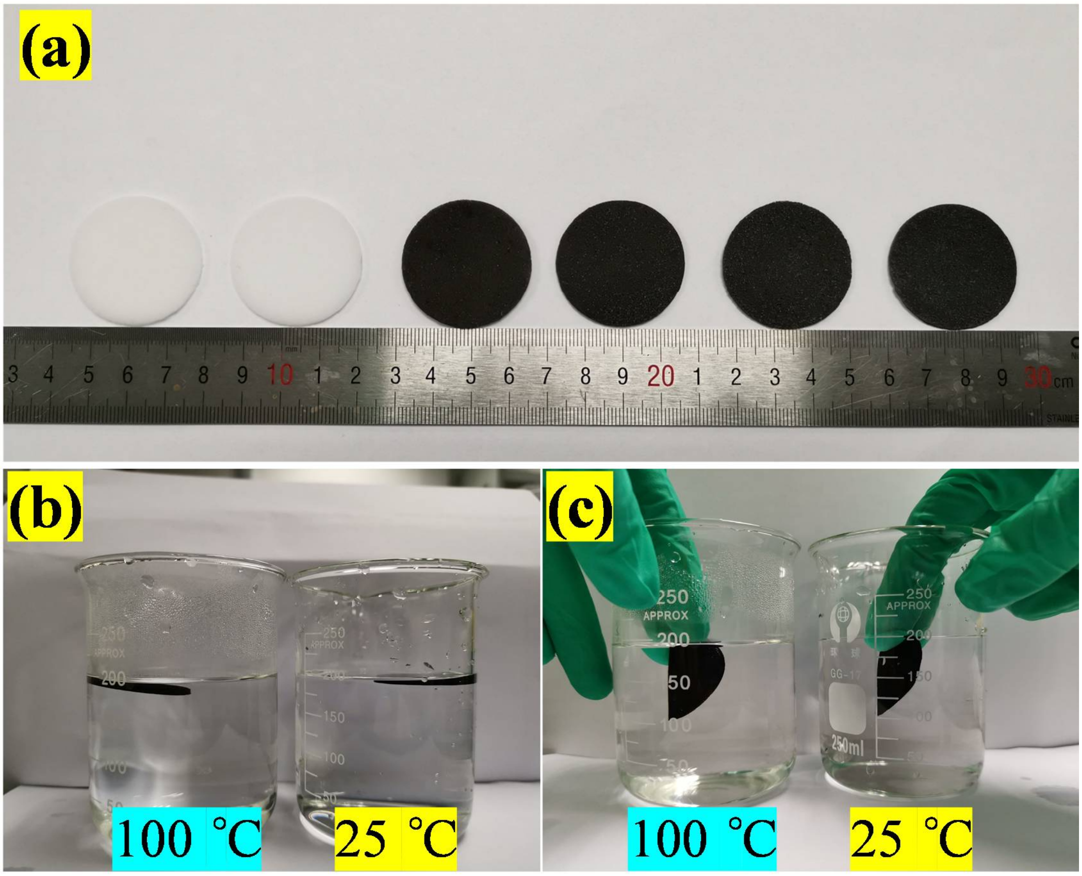

Fig. S2 (a) Photographs of neat MS, PDDA@MS, Te-Se@PDDA@MS with Te-Se loadings of 39.3 wt%, 74.1 wt%, 103.4 wt% and 133.4 wt%, respectively (from left to right); The free states (b) and bending states (c) of Te-Se@PDDA@MS with a Te-Se loading of 133.4 wt% in boiling water and room-temperature water.

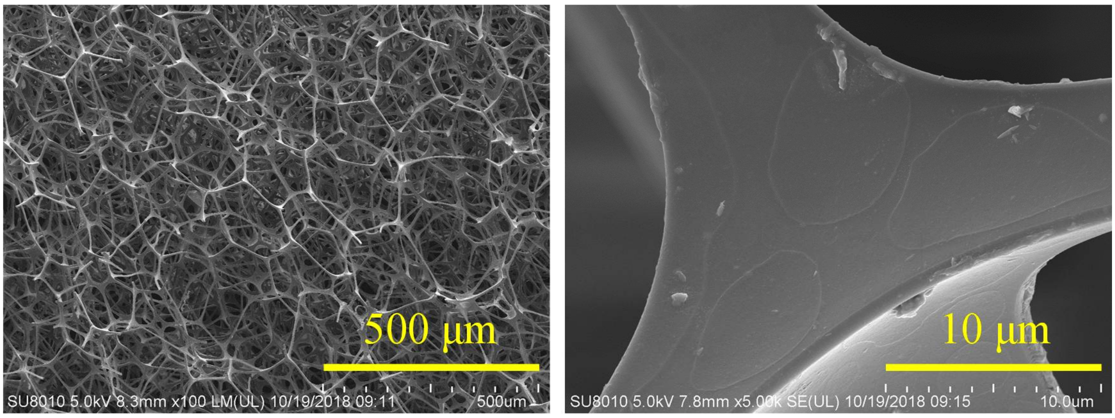

Fig. S3 SEM images illustrating the surface morphology of neat MS without any treatment.
